# Amyloid Fibers of α-Synuclein Catalyze Chemical Reactions

**DOI:** 10.1021/acschemneuro.2c00799

**Published:** 2023-02-06

**Authors:** Istvan Horvath, Pernilla Wittung-Stafshede

**Affiliations:** Department of Biology and Biological Engineering, Chalmers University of Technology, 412 96 Gothenburg, Sweden

**Keywords:** Catalysis, amyloid fibers, α-synuclein, Parkinson’s disease, catalytic efficiency

## Abstract

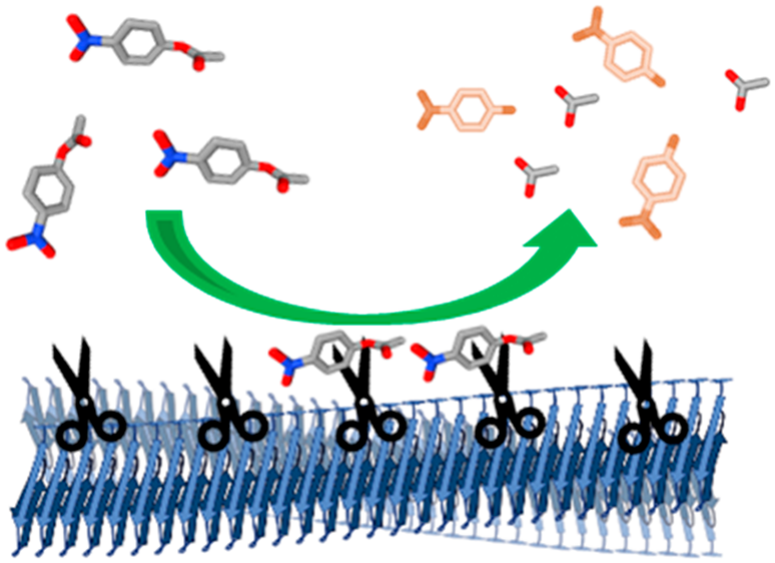

Amyloid fibers of the protein α-synuclein, found in Lewy body deposits, are hallmarks of Parkinson’s disease. We here show that α-synuclein amyloids catalyze biologically relevant chemical reactions in vitro. Amyloid fibers, but not monomers, of α-synuclein catalyzed hydrolysis of the model ester *para*-nitrophenyl acetate and dephosphorylation of the model phosphoester *para*-nitrophenyl-orthophosphate. When His50 was replaced with Ala in α-synuclein, dephosphorylation but not esterase activity of amyloids was diminished. Truncation of the protein’s C-terminus had no effect on fiber catalytic efficiency. Catalytic activity of α-synuclein fibers may be a new gain-of-function that plays a role in Parkinson’s disease.

Amyloid fibrils are polymers of monomeric protein units noncovalently assembled through β-strands arranged perpendicularly to the fibril axis forming a cross-β structure.^1^ Many proteins can form amyloid fibrils at certain solvent conditions in vitro,^1^ but this process is mostly connected to human neurodegenerative diseases such as Alzheimer’s disease and Parkinson’s disease (PD).^2−4^ In these diseases, the amyloid fibrils are often considered end products with intermediate species (so-called oligomers) formed during aggregation as most toxic to cells. Deleterious gain-of-functions coupled to amyloid assembly are mitochondrial dysfunction, oxidative stress, protein degradation failure, and eventually cell death.^5^ In this work, we demonstrate a new functionality for the amyloid fibers found in PD that challenge the concept of pathological amyloids as inert species.

PD is the second most common neurodegenerative disorder and the most frequent movement disorder today for which there is only symptomatic treatment.^6,7^ Amyloid fibers of the protein α-synuclein constitute the major content of pathological intraneuronal inclusions, Lewy bodies, found in PD patient brains.^8−10^ In accord, duplications, triplications, and point-mutations in the α-synuclein gene, enhancing expression and aggregation, are linked to familial PD cases.^11^ Although soluble α-synuclein oligomers are proposed to be most toxic,^12,13^ it is clear that α-synuclein amyloid fibrils themselves are toxic too and can be transmitted from cell to cell and cross the blood-brain barrier.^14−16^ The core β-structure of α-synuclein amyloid fibers is hydrophobic and involves approximately residues 60–94 (varies between different structures reported; most often, the amyloid fold starts at His50 or a few residues earlier). The N- and C-termini stretches protrude, as a fuzzy coat, from the ordered core: residues 1 to 60 are amphipathic with many basic residues, whereas residues 95 to 140 are acidic with many negatively charged residues (Figure S1).

Intriguingly, it was recently reported that amyloid fibers of the Alzheimer’s disease peptide amyloid-β (Aβ)^17^ and of the glucose-regulating hormone glucagon,^18^ but not the monomeric counterparts, catalyzed pathological and metabolic chemical transformations in vitro. It was proposed that the amyloid fibers, due to their polymeric nature, display distinct catalytic sites at the fibril’s surface.^19^ To assess the generality of these findings, we set out to probe if α-synuclein amyloid fibers harbor catalytic, enzyme-like properties toward biological reactions.

Initial amyloid fibers of wild-type (and mutants, see below) α-synuclein were made from monomers in the presence of beads and agitation. These amyloids were then used to seed new monomers to form amyloid fibers at quiescent conditions (resulting in more homogeneous amyloids). Amyloids formed in this 2-step way were used as the starting point in seeding reactions to create fresh amyloids for each set of experiments. The formed α-synuclein amyloids were characterized with atomic force microscopy (AFM), and the α-synuclein variants used here all formed amyloids that appeared similar (Figure S2). We first tested for esterase activity of wild-type α-synuclein amyloids using hydrolysis of *para*-nitrophenyl acetate (pNPA) as substrate. Formation of the hydrolysis phenol product (pNP) can readily be detected via absorption at 410 nm (Figure 1A). In Figure 1, we show kinetic time traces at different substrate concentrations (B), initial rates (*V*_o_) as a function of wild-type α-synuclein fiber concentration (C), and *V*_o_ as a function of substrate concentration for a fixed α-synuclein fiber concentration (10 μM) (D). Based on the data, monomers harbor almost no activity, whereas the amyloids do. A plot of *V*_o_ versus α-synuclein amyloid concentration is linear. Notably, the amount of converted substrate is multiple times higher than the α-synuclein concentration used in the assays, attesting to catalytic activity of the fibers. A nonlinear fitting of the data in Figure 1D to the Michaelis–Menten reaction model provides *K*_M_ and *k*_cat_ values for the reaction. With *K*_M_ (Michaelis constant) of 4.3 mM and *k*_cat_ (turnover number) of 0.011 s^–1^ giving the catalytic efficiency ε = *k*_cat_/*K*_M_ of 2.9, the α-synuclein amyloids exhibit higher catalytic efficiency toward pNPA than reported for Aβ amyloids (ε = 0.64)^17^ (Table 1).

**Figure 1:**
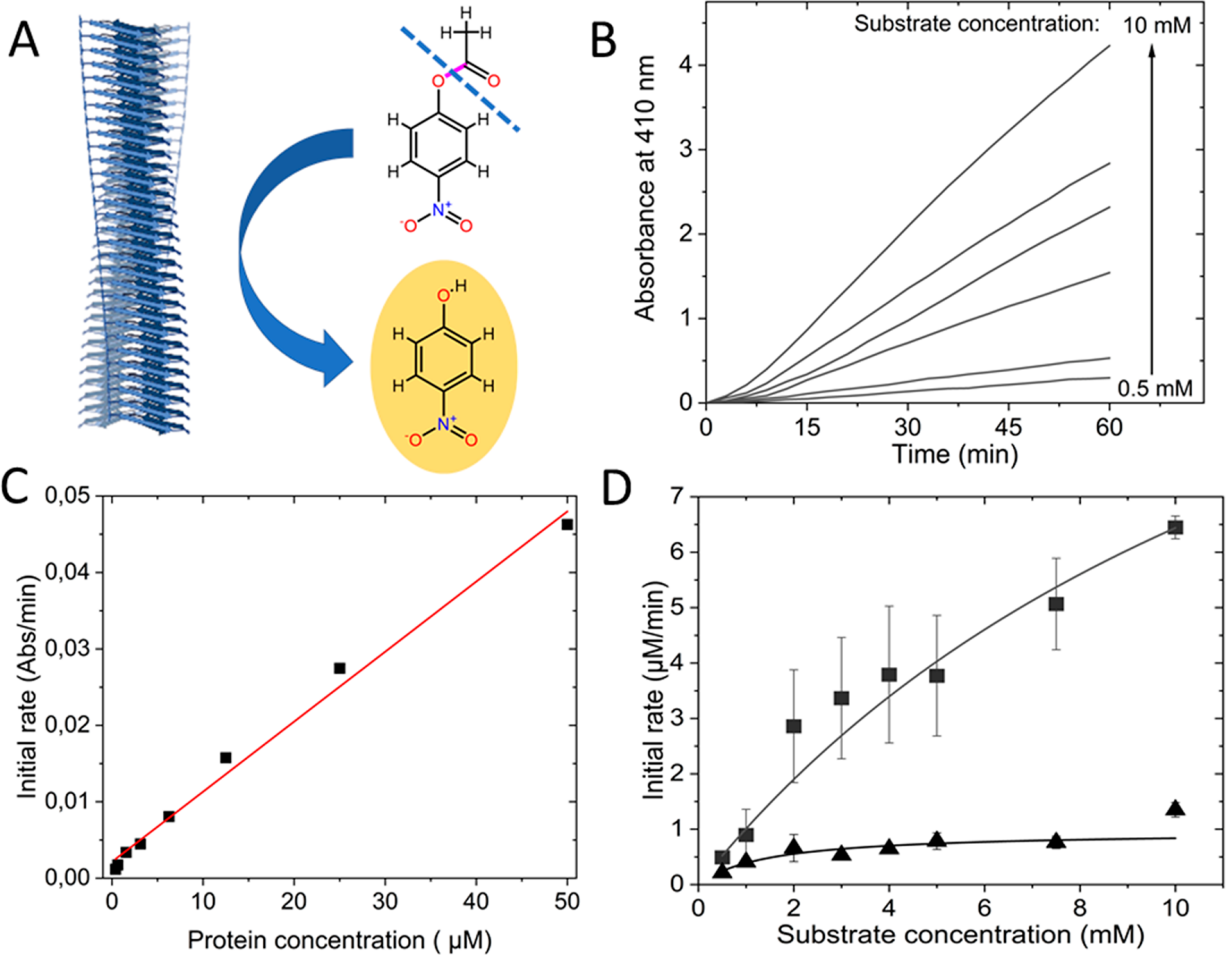
**Esterase activity**. (A) Scheme of the chemical reaction probed in this assay, pNPA to pNP, with the C–O bond that is cleaved highlighted in purple and dashed line. (B) Kinetic traces of pNP build up as followed by absorbance at 410 nm at various initial pNPA concentrations in the presence 10 μM α-synuclein amyloid fibers. (C) Initial rates of esterase activity as a function of α-synuclein amyloid fiber concentration measured with 2 mM pNPA as substrate. (D) Michaelis–Menten plot of initial reaction rates as a function of pNPA concentrations in the presence of 10 μM amyloid fiber (■, with Michaelis–Menten fit as solid curve) or monomeric (▲) α-synuclein. (*N* = 3, *R*^2^_monomer_ = 0.86, *R*^2^_fiber_ = 0,96).

**Table 1:** Michaelis-Menten Enzymatic Parameters (*K*_M_, *K*_cat_, ε) Determined for Catalytic Activity (Esterase and Phosphatase) of Amyloid Fibers (Amyloid Fibers of Three α-Synuclein Variants, This Work; Aβ and Glucagon Amyloid Fiber Data Taken from Published Work)^a^

		α-synuclein amyloid fibers				
		WT	α-synuclein H50A	α-synuclein (1–119)	Aβ fibers^b^	Glucagon fibers^c^
esterase	*K*_M_ (mM)	4.3 ± 2.5	3.6 ± 2.4	5.2 ± 1.5	2.9	4.4
	*k*_cat_ (s^–1^)	0.012 ± 0.003	0.011 ± 0.003	0.011 ± 0.002	0.0019	0.0025
	ε	2.9	2.8	2.1	0.64	0.55
phosphatase	*K*_M_ (mM)	0.5 ± 0.13	1.2 ± 2.3	0.4 ± 0.12	*n.d*.	0.12
	*k*_cat_ (s^–1^)	0.0003 ± 0.0001	6 × 10^–5^ ± 1 × 10^–5^	0.0003 ± 0.0001	*n.d*.	0.0070
	ε	0.6	0.05	0.7	*n.d*.	57

a *n.d*. not determined. See Figures 1, 2, and S3 for graphs of α-synuclein data. Monomers of the α-synuclein variants showed no catalytic activity in any of the assays.

b From ref (17). Conditions: 50 mM HEPES, pH 7.35, 37 °C.

c From ref (18). Conditions: 50 mM HEPES, pH 7.4, 37 °C.

When we repeated the same experiments with α-synuclein amyloids made from α-synuclein variants with His50 exchanged for Ala (α-synuclein His50Ala) or with the C-terminal 21 residues truncated (α-synuclein(1–119)), the same catalytic result was observed: amyloids catalyzed esterase activity similarly to wild-type α-synuclein fibers, whereas monomers did not (Figure 1, Table 1, Figure S3).

We also tested if α-synuclein amyloids (wild-type and the variants α-synuclein His50Ala and α-synuclein(1–119)) had phosphatase activity using *para*-nitrophenyl orthophosphate (pNPP) as substrate (Figure 2A). Figure 2 shows kinetic time traces (detecting product formation at 410 nm) at different substrate concentrations (B), initial rates (*V*_o_) as a function of α-synuclein fiber concentration (C), and resulting *V*_o_ versus substrate concentration Michaelis–Menten plots for wild-type and H50A amyloid fiber variants (D). Dephosphorylation activity was not assessed for Aβ amyloids but reported for glucagon amyloid fibers.^18^ When the Michaelis–Menten parameters are compared to those reported for glucagon amyloids, the catalytic efficiency of α-synuclein fibers is several orders of magnitude lower (Table 1). Still, wild-type and α-synuclein(1–119) amyloids exhibited phosphatase activity rather similar in magnitude to that detected for α-synuclein-mediated esterase activity. In contrast, monomers had no catalytic activity, and, notably, when His50 was replaced with Ala, the resulting α-synuclein His50Ala amyloids had negligible activity (Figure 2D, Table 1). Thus, for dephosphorylation, the sole His residue in α-synuclein, positioned in, or at the edge of, the ordered amyloid core (Figure S4), appears to play a key role. In support, His1 in glucagon was noted as very important for the high phosphatase activity found for glucagon amyloids.^18^

**Figure 2:**
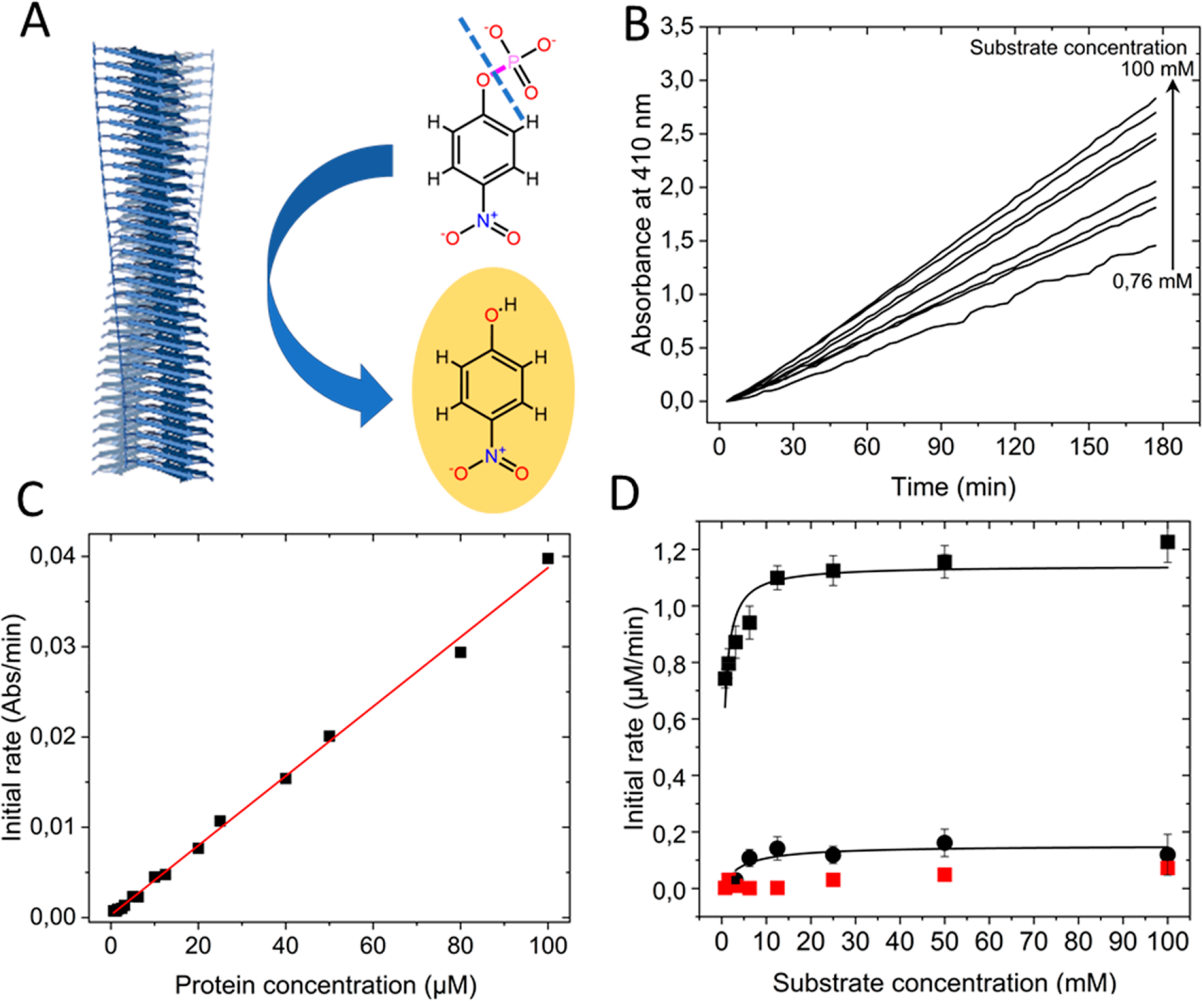
**Phosphatase activity**. (A) Scheme of the chemical reaction probed in this assay, pNPP to pNP, with the P–O bond that is cleaved highlighted in purple and dashed line. (B) Kinetic traces of pNP build up as followed by absorbance at 410 nm at various initial pNPP concentrations in the presence 40 μM aS amyloid fibers. (C) Initial rates of phosphatase activity as a function of α-synuclein amyloid fiber concentration measured with 5 mM pNPP as substrate. (D) Michaelis–Menten plot of initial reaction rates as a function of pNPP concentrations in the presence of 40 μM wild-type amyloid fiber (black squares, with Michaelis–Menten fit as solid curve), His50Ala amyloid fiber (black spheres), or wild-type monomeric (red squares) α-synuclein. (*N* = 3, *R*^2^_WTfiber_ = 0.83, *R*^2^_H50Afiber_ = 0.64).

Synthetic peptides forming amyloids have been designed to display catalytic domains on the amyloid surface,^19,20^ and self-assembled peptides have been implicated as primitive enzymes in the prebiotic world.^21^ This study demonstrates that α-synuclein amyloid fibers—hallmarks of PD—harbor distinct catalytic activity in vitro. The amyloid fiber structure appears as a crucial scaffold, as monomers had little or no enzymatic activity. The repetitive arrangement of surface-exposed residues, which can act as nucleophiles, in the amyloid structure may create weak-affinity substrate binding sites. Likely, residues such as Lys, Ser, Tyr, and Thr, along with His, play important roles in forming such catalytic sites,^19^ as shown for small synthetic peptide amyloids.^22^ Catalytic triads (nucleophile, acid and base residues) often form the active sites in enzymes that cleave chemical bonds. In similarity to this, α-synuclein has many Asp and Glu residues (can act as acid), in addition to His50, many Lys residues (acting as base), and a number of Ser and Thr residues (can act as nucleophile); several such side chains are placed in small clusters on the outside of the amyloid core and may create active sites (Figure S4). Further molecular-mechanistic studies are desired to elucidate structure–function relationships for this new amyloid functionality.

Chemical catalysis of amyloid fibers (Figure 3), reported here for α-synuclein and previously for Aβ,^17^ underscore the possible existence of yet unexplored pathological pathways associated with amyloids in neurodegenerative disorders such as Alzheimer’s and PD. Metabolic changes have been detected in samples from PD patients^23^ and, cell culture experiments, where cells have been challenged with amyloidogenic proteins, have shown alterations in metabolites.^23−25^ One may also imagine that physiological amyloids, such as the pigment cell-specific premelanosomal protein (PMEL) amyloids, not only act as a scaffold for highly reactive melanin intermediates but, perhaps, also catalyze the chemical reactions that result in polymerized melanin pigment.^26^ To conclude, our results presented here suggest that catalytic activity of amyloid fibers may be a new gain-of-function feature of pathological aggregates that is responsible for (some of) the detected metabolite alterations found in neurodegenerative disorders. Catalytic ability may also be an additional functionality of various functional amyloid systems (that often play structural roles) not yet explored.

**Figure 3:**
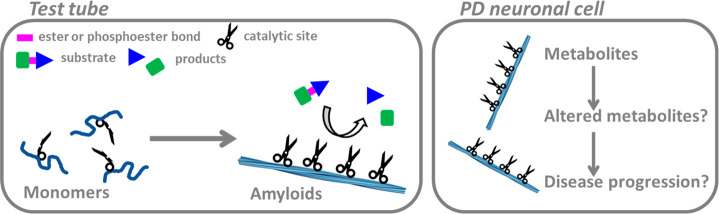
**Schematic illustration of catalytic α-synuclein amyloids in vitro and in vivo**. In test tube experiments (left), α-synuclein monomers exhibit no catalytic activity (depicted as only half scissor), whereas upon assembly to amyloid fibers (depicted as formation of functional pairs of scissors), catalysis of ester and phosphoester bonds is observed. We propose (right) that catalytic activity of α-synuclein amyloid fibers in neuronal cells of PD patients may contribute to altered metabolism that, in turn, may further stimulate disease progression.

## Methods

### Protein Expression and Purification

Wild-type (WT), His50Ala, and 1–119 (truncated after residue 119) α-synuclein protein was expressed in *Escherichia coli* grown in Luria broth (LB) and purified using anion exchange chromatography and gel filtration as previously reported.^27^ The purified protein aliquots were stored at −80 °C. Before use, gel filtration was performed to obtain homogeneous monomeric α-synuclein solution using a Superdex 75 10/300 (Cytiva, Uppsala, Sweden) column in Tris-buffered saline (TBS) buffer (50 mM Tris, 150 mM NaCl, pH 7.6 at 25 °C, Medicago AB, Uppsala, Sweden).

### Preparation of α-synuclein Amyloid Fibers

Freshly gel-filtered 100 μM α-synuclein variant (WT, His50Ala or 1–119 α-synuclein) was incubated with agitation using glass beads at 37 °C in TBS in a plate reader incubator (Figure S2 top). Under these conditions amyloid formation of the α-synuclein proteins is complete after less than 72 h. After 3 days of incubation, the aggregated protein was added to 250 μM fresh monomeric α-synuclein protein so that the concentration of preaggregated protein was 5% of the monomeric protein. The mixture was incubated for 5 days at 37 °C. During the incubation the initially transparent protein solutions turned turbid, which is indicative of seeded amyloid formation. Following the completion of this seeded aggregation, the samples were aliquoted, flash frozen in liquid nitrogen, stored at −80 °C, and used as premade stock amyloids. For the preparation of fresh amyloid fibers used in the activity measurements, a stock of premade amyloid fibers from −80 °C was added to freshly gel-filtered monomeric protein (∼250 μM) at 5% ratio and incubated for 5 days at 37 °C. At the end of the incubation, samples were centrifuged at 13 500*g* for 30 min to separate amyloid fibers from remaining monomers and smaller assemblies. The pellet was resuspended in TBS, and the protein concentration of the pellet fraction was approximated from the protein concentration in the supernatant measured by absorbance at 280 nm.

### Esterase Assay

*para*-Nitrophenyl acetate (pNPA) (Sigma-Aldrich) was dissolved in acetonitrile (Sigma-Aldrich) at a concentration of 100 mM. The protein solutions were diluted into phosphate buffer (50 mM, pH 7.0), and the required amount of pNPA was added to the protein (monomeric or amyloid fiber forms of α-synuclein variants) solution. Blank measurements were performed by omitting the protein from samples but using the same volume of TBS buffer mixed into the phosphate buffer. The corresponding blank measurements were subtracted from the absorbance values obtained in the presence of added protein. An example of blank measurements with each substrate is shown in the Supporting Information (Figure S5). The samples were incubated in 96-well, half area transparent-bottom plates with a nonbinding surface (CLS3881; Corning, Corning, NY) at 37 °C using a plate reader incubator instrument (Fluorostar Optima; BMG Labtech, Ortenberg, Germany). Absorbance at 410 nm was measured every 3 min over 60 min. The initial rate calculation was performed on data points between 10 and 40 min. The extinction coefficient of 7500 M^–1^ cm^–1^ (for *para*-nitrophenyl, pNP, in 50 mM phosphate, pH 7) was used to relate absorption to product concentration and calculate initial rates. Each experiment included 3 technical replicates for each condition, and at least 3 independent experiments were performed for each. The reported kinetic parameters in Table 1 are the average of at least 3 independent experiments (*N*), while kinetic curves shown in Figure 1 are representative independent experiments with 3 technical replicates.

### Phosphatase Assay

*para*-Nitrophenyl-ortophosphate (pNPP) (Sigma-Aldrich) was dissolved in Milli-Q water at a concentration of 200 mM. The reaction buffer was TBS with added 1 mM ethylenediaminetetraacetic acid (EDTA), pH 7.4. The preparation of the samples and measurements were performed similarly to those for the esterase assay. For the calculation of initial rates, an extinction coefficient of 13 500 M^–1^ cm^–1^ for pNP (TBS, pH 7.4) at 410 nm was used. Each experiment included 3 technical replicates for each condition, and at least 3 independent experiments were performed for each. The reported kinetic parameters in Table 1 are the average of at least 3 independent experiments (*N*), while kinetic curves shown in Figure 2 are representative independent experiments with 3 technical replicates.

### Atomic Force Microscopy (AFM)

Amyloid fibers were diluted 20 times into Milli-Q water and deposited on freshly cleaved mica. After 10 min, the mica was rinsed with filtered Milli-Q water and dried under a gentle nitrogen stream. Images were recorded on an NTEGRA Prima setup (NT-MDT, Moscow, Russia) using a gold-coated single-crystal silicon cantilever (NT-MDT, NSG01, spring constant of ∼5.1 N/m) and a resonance frequency of ∼180 kHz in tapping mode. 512 × 512-pixel images were acquired with a scan rate of 0.5 Hz. Images were analyzed using the WSxM 5.0 software.^28^

## Supporting Information Available

Figures S1–S5, showing α-synuclein amino acid sequence and amyloid fold (S1), characterization of aggregation process and resulting amyloids of α-synuclein variants used (S2), additional Michaelis–Menten plots (S3), α-synuclein amyloid structure with His50 and putative catalytic sites indicated (S4), and background traces of the kinetic assays (S5) (PDF)
