# Supplementary material for: Amyloid Fibers of α-Synuclein Catalyze Chemical Reactions
Source: ACS Chem Neurosci. 2023 Feb 6;14(4):603–8. doi: 10.1021/acschemneuro.2c00799 (PMC9936539; doi:10.1021/acschemneuro.2c00799)
Supplement: Supplementary file 1 — cn2c00799_si_001.pdf [file cn2c00799_si_001.pdf]

Supporting Information

Amyloid fibers of α-synuclein catalyze chemical reactions

Istvan Horvath and Pernilla Wittung-Stafshede

Department of Biology and Biological Engineering, Chalmers University of Technology, 412 96 Gothenburg, Sweden

Content:

Figures S1-S5

MDVFMKGLSK AKEGVVAAAE KTKQGVAEAA GKTKEGVLYV GSKTKEGVVH
GVATVAEKTK EQVTNVGGAV VTGVTAVAQK TVEGAGSIAA ATGFVKKDQL
GKNEEGAPQE GILEDMPVDP DNEAYEMPSE EGYQDYEPEA

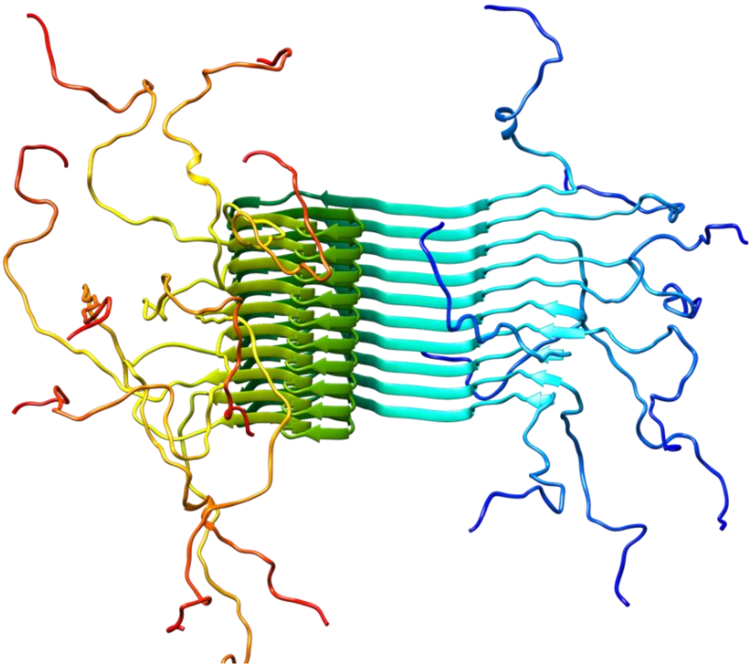

Figure S1.
Amino acid sequence of aS monomer, with residues colored according to the Lesk color code (top). His50, underlined. Arrow indicates the truncation site in the aS(1-119) variant. Pdb structure of full-length aS amyloid fiber (2N0A), illustrating the floppy N- and C-termini (bottom).

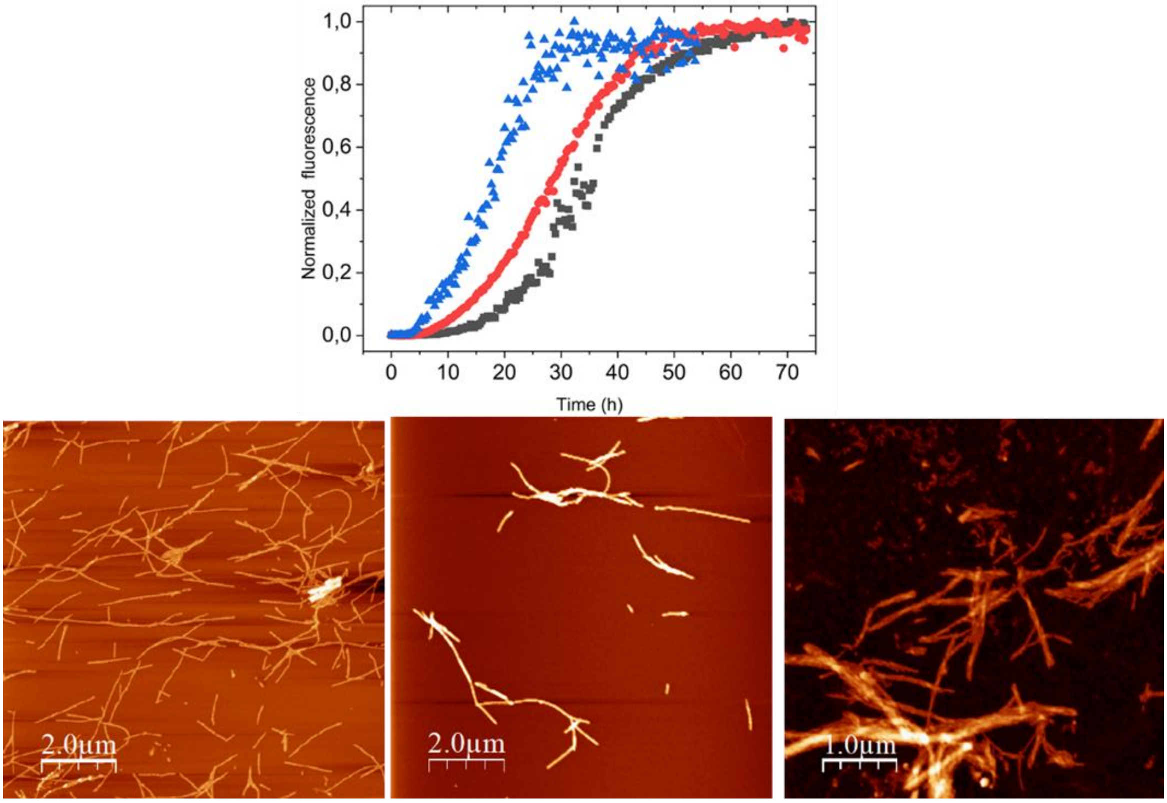

Figure S2.

Top. Thioflavin-T fluorescence monitored amyloid formation reactions of the three aS variants used in this study (wild type, black; His50Ala aS, red; aS 1-119, blue), showing that they all readily aggregate (beads, agitation, TBS, 37 °C, 100 μM protein monomers). **Bottom.** AFM of amyloid fibers of (from left to right) wild-type, His50Ala and 1-119 aS, revealing visually similar amyloids although the truncated variant amyloids appear to cluster more than the others.

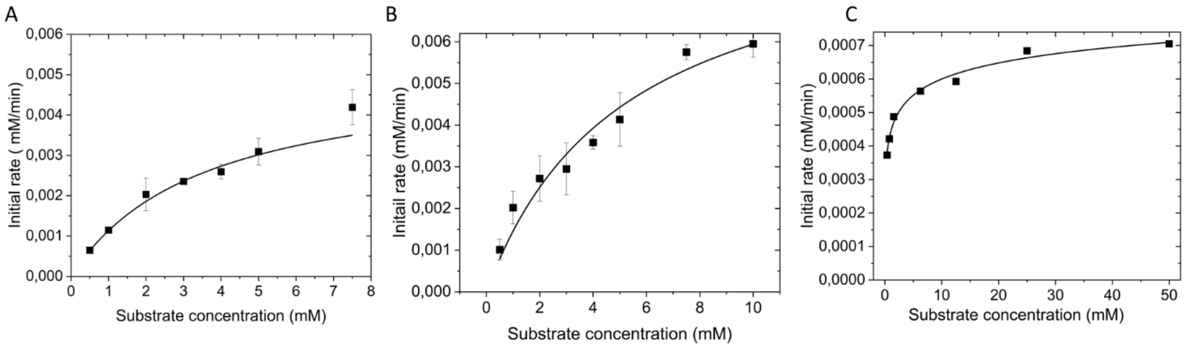

Figure S3.
Additional Michaelis-Menten plots, complementing **Figures 1** and **2** and parameters listed in **Table 1**. **A. Esterase.** Michael-Menten plot of initial reaction rates as a function of pNPA concentrations in the presence of 10 µM His50Ala aS amyloid fiber (squares, with Michaelis-Menten fit as solid curve). **B. Esterase.** Michael-Menten plot of initial reaction rates as a function of pNPA concentrations in the presence of 10 µM aS(1-119) amyloid fiber (squares, with Michaelis-Menten fit as solid curve). **C. Phosphatase.** Michaelis-Menten plot of initial reaction rates as a function of pNPP concentrations in the presence of 40 µM aS(1-119) amyloid fiber (black squares, with Michaelis-Menten fit as solid curve).

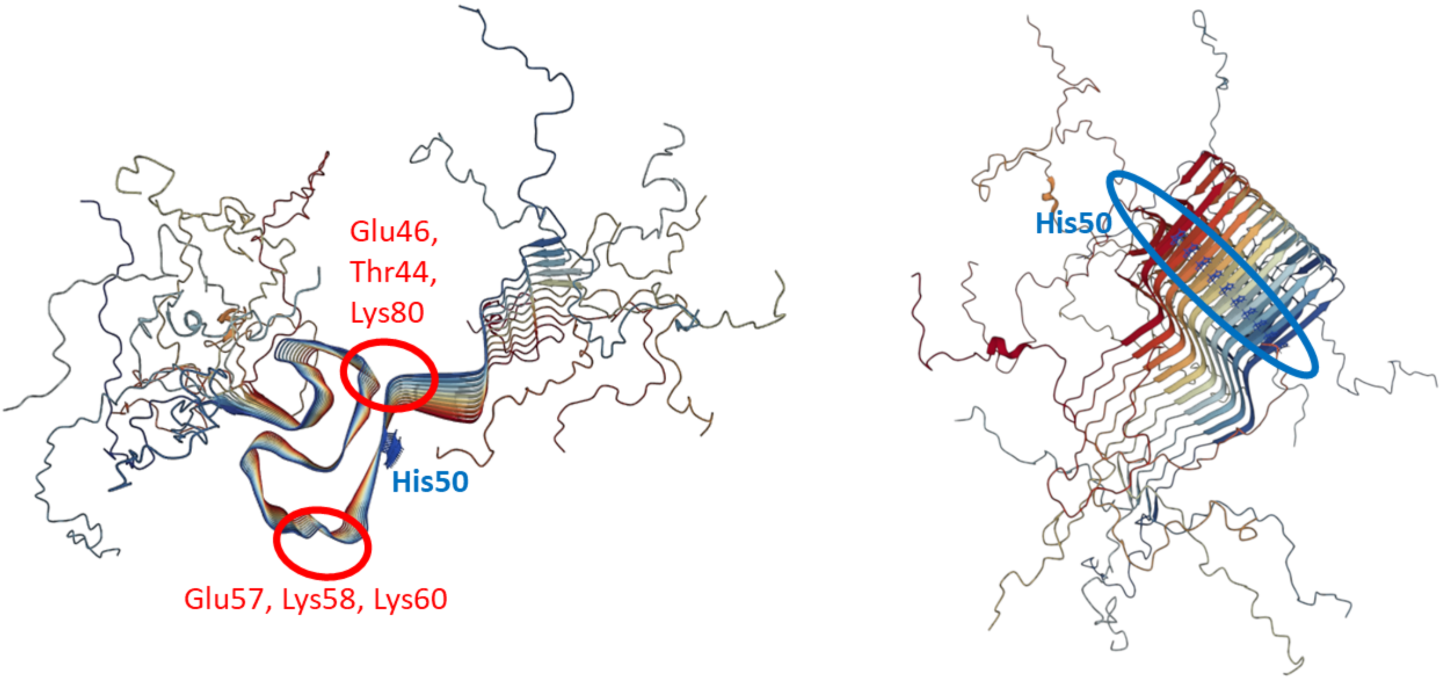

Figure S4.
Pdb structure of full-length aS amyloid fiber (2N0A), showing the floppy N- and C-termini in two views. His50 is here highlighted in stick representation. We note that there are several pdb structures of aS amyloids with differences in the exact Greek-key fold. In most aS amyloid structures, His50 is within or at the edge of the folded core, with its side chain facing outwards. Two areas are circled in red as illustration of two regions with clustered residues that may contribute to catalytic activity. There are more places for possible catalytic activity along the amyloid core surface, and it is possible that residues from the floppy ends contribute by interacting inwards to the amyloid fiber surface. In addition, individual amyloid fibers pack with each other to form mature amyloid fibers and thus there may also be active sites formed between proximal residues in different amyloid fibers. We note the website https://people.mbi.ucla.edu/sawaya/amyloidatlas/ that includes all known structures of aS amyloids (as of January 2023) where one can easily identify His50 and its surroundings in each reported structure.

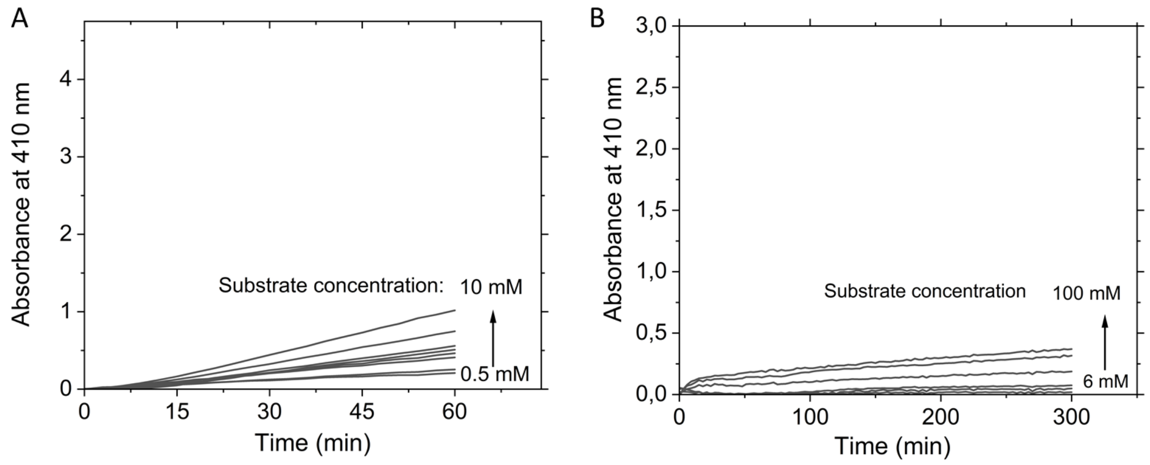

Figure S5.
Background measurements performed in the absence of added protein in the esterase (A) and phosphatase (B) assays at various concentrations of the respective substrates. The Y axis scale is set to the same limits as in **Figures 1** and **2** for easier comparison.
